# Evaluating the role of *NTHL1* p.Q90* allele in inherited breast cancer predisposition

**DOI:** 10.1002/mgg3.1493

**Published:** 2020-09-19

**Authors:** Timo Kumpula, Anna Tervasmäki, Tuomo Mantere, Susanna Koivuluoma, Laura Huilaja, Kaisa Tasanen, Robert Winqvist, Richarda M. de Voer, Katri Pylkäs

**Affiliations:** ^1^ Laboratory of Cancer Genetics and Tumor Biology University of Oulu Oulu Finland; ^2^ Department of Human Genetics Radboud University Medical Center Nijmegen Netherlands; ^3^ Department of Dermatology and Medical Research Center Oulu PEDEGO Research Unit University of Oulu Oulu University Hospital Oulu Finland; ^4^ Department of Human Genetics Radboud Institute for Molecular Life Sciences Radboud University Medical Center Nijmegen Netherlands

## Abstract

**Background:**

Rare protein truncating variants of *NTHL1* gene are causative for the recently described, recessively inherited *NTHL1* tumor syndrome that is characterized by an increased lifetime risk for colorectal cancer, colorectal polyposis, and breast cancer. Although there is strong evidence for breast cancer being a part of the cancer spectrum in these families, the role of pathogenic *NTHL1* variants in breast cancer susceptibility in general population remains unclear.

**Methods:**

We tested the prevalence of *NTHL1* nonsense variant c.268C>T, p.Q90*, which is the major allele in *NTHL1* families and also shows enrichment in the Finnish population, in a total of 1333 breast cancer patients. Genotyping was performed for DNA samples extracted from peripheral blood by using high‐resolution melt analysis.

**Results:**

Sixteen *NTHL1* p.Q90* heterozygous carriers were identified (1.2%, *p* = 0.61): 5 in hereditary cohort (n = 234, 2.1%, *p* = 0.39) and 11 in unselected cohort (n = 1099, 1.0%, *p* = 0.36). This frequency is equal to that in the general population (19/1324, 1.4%). No *NTHL1* p.Q90* homozygotes were identified.

**Conclusion:**

Our results indicate that *NTHL1* p.Q90* heterozygous carriers do not have an increased risk for breast cancer and that the variant is unlikely to be a significant contributor to breast cancer risk at the population level.

## Abstract 2

Although there is strong evidence for breast cancer being a part of the cancer spectrum of recessively inherited NTHL1 tumor syndrome, the role of pathogenic *NTHL1* variants in breast cancer susceptibility in the general population remains unclear. Here, we have tested the prevalence of *NTHL1* nonsense variant c.268C>T, p.Q90*, which is the major allele in NTHL1 families and also shows enrichment in the Finnish population, in a total of 1333 breast cancer patients. Our results indicate that *NTHL1* p.Q90* heterozygous carriers do not have an increased risk for breast cancer and that the variant is unlikely to be a significant contributor to breast cancer risk at the population level, a notion that is particularly important for genetic counseling.

## 1 INTRODUCTION

Base excision repair (BER) is the main mechanism for repairing endogenous DNA damage that results from oxidation, deamination, depurination, and alkylation, thereby protecting the genome from mutations (Krokan & Bjørås, 2013). Biallelic germline loss‐of‐function variants in the BER pathway initiating DNA glycosylase (Limpose et al., 2018), *NTHL1* (OMIM *602656), were recently reported to underlie a novel recessive adenomatous polyposis and colorectal cancer predisposition syndrome (OMIM #616415) (Kuiper & Hoogerbrugge, 2015; Weren et al., 2015). The most prevalent *NTHL1* variant in these polyposis families was the nonsense variant p.Q90* (c.268C>T, rs150766139), which to date has been reported in 18 families (Grolleman et al., 2019; Kuiper & Hoogerbrugge, 2015; Weren et al., 2015). Studies on the polyposis families suggested that besides polyposis/colorectal cancer, also the risk for various other cancer types, including breast cancer, is increased. Grolleman et al. (2019) provided additional support for this by reporting women with biallelic deleterious *NTHL1* variants having an unexpectedly high breast cancer incidence (60%, 9 out of 15 of the studied cases). While the increased cancer risk is established for individuals with biallelic *NTHL1* pathogenic variants, the risk estimates for heterozygous carriers are unclear (Grolleman et al., 2019).

According to public databases, the *NTHL1* p.Q90* allele is enriched in the Finnish population (Finnish minor allele frequency [MAF] 0.0038 versus global MAF 0.0014) with a carrier frequency of 19/1324 (1.4%, MAF 0.007) in North Ostrobothnia (gnomAD, https://gnomad.broad​insti​tute.org/ (Karczewski et al., 2020); SISu, http://www.sisup​roject.fi/). This geographical enrichment provides an excellent opportunity to test the association of *NTHL1* p.Q90* with breast cancer susceptibility at the population level, along with the potential to establish risk estimates for the allele at heterozygous state. For this purpose, here, we have tested the prevalence of *NTHL1* p.Q90* in breast cancer patients with an indication of hereditary disease susceptibility and those unselected for the family history of cancer and age at disease onset, all collected from the North Ostrobothnia area.

## 2 MATERIALS AND METHODS

### 2.1 Ethical compliance

This study included informed consent from all participating individuals, and it was approved by the Ethical Board of the North Ostrobothnia Health Care District.

### 2.2 Breast cancer cohorts

The hereditary cohort (n = 234), collected from the North Ostrobothnia area (Oulu University Hospital), included *BRCA1*/*BRCA2*/*PALB2* mutation‐negative breast cancer cases with the indication of an inherited predisposition to the disease. Cases were selected using the following criteria: 1) index cases from families with three or more breast and/or ovarian cancer cases in first‐ or second‐degree relatives (n = 125), and 2) index cases from families with two cases of breast, or breast and ovarian cancer in first‐ or second‐degree relatives, of which at least one with early disease onset (<35 years), bilateral disease or multiple primary tumors (n = 29), and 3) breast cancer cases diagnosed at or below the age of 40 (n = 80). The young breast cancer cases were included based on the assumption that when a woman below the age of 40 years develops breast cancer, a hereditary predisposition can be suspected regardless of the family history (Brunet, 2010). The unselected breast cancer cohort consisted of 1099 consecutive breast cancer cases diagnosed at the Oulu University Hospital during the years 2000–2016 (with a mean age of 58 years at diagnosis) and were unselected for the family history of cancer and age at disease onset.

### 2.3 Variant detection

Genotyping was performed for DNA samples extracted from peripheral blood by using high‐resolution melt analysis (CFX96, Bio‐Rad) with Type‐It HRM reagents (Qiagen). All assays included heterozygous and homozygous *NTHL1* p.Q90* (NM_002528.5) genomic DNA samples as positive controls. Verification of all detected p.Q90* variants were confirmed with Sanger sequencing (ABI3130xl, Applied Biosystems).

### 2.4 Statistical analyses

Fisher's exact test was used to compare the carrier frequency between cases and controls, and Mann–Whitney *U* test to compare the mean age at diagnosis between carrier and noncarrier cases in unselected cohort (IBM SPSS Statistics 24.0 for Windows, IBM Corp.). All *p* values were two‐sided and values <0.05 were considered statistically significant.

## 3 RESULTS

Five cases from the hereditary cohort were identified as heterozygous *NTHL1* p.Q90* carriers (5/234, 2.1%, *p* = 0.39, odds ratio [OR] = 1.5, 95% confidence interval [CI] = 0.6–4.1, Table 1). The presence of other pathogenic germline *NTHL1* variants in them was ruled out by targeted gene panel sequencing. The carriers were diagnosed with breast cancer at the age of 34, 38, 38, 47, and 49 years, respectively. In these families, two additional breast cancer cases (family members of Her3 and Her4, respectively) were available for testing and one of them (breast cancer at the age of 62 years) was identified to carry *NTHL1* p.Q90* (Table 2). In the family of index Her1, one case with stomach cancer and one with uterus cancer were also identified as carriers, whereas the salivary gland cancer case tested negative. In two other families (index Her3 and Her5, Table 2), DNA samples from altogether four family members diagnosed with other cancer types (basal cell carcinoma, prostate cancer, adenocarcinoma, and renal cancer, respectively) were available for testing, but all turned out as noncarriers. Based on these analyses, the evidence for *NTHL1* p.Q90* segregating with cancer within these families remains uncertain.

**TABLE 1:** Frequency of *NTHL1* p.Q90* (c.268C>T)^a^ in the studied breast cancer cohorts and controls.

Cohort	N	WT	%	Mut^b^	%	OR	95% CI	*p* c
Hereditary	234	229	97.9	5	2.1	1.5	0.6–4.1	0.39
Unselected	1099	1088	99.0	11	1.0	0.7	0.3–1.5	0.36
All BC	1333	1317	98.8	16	1.2	0.8	0.4–1.6	0.61
Controls (SISu)^d^	1324	1305	98.6	19	1.4			

Abbreviations: BC, breast cancer; CI, confidence interval; Mut, mutation; OR, odds ratio; WT, wild type.

a GenBank reference sequence NM_002528.5.

b All heterozygous.

c Fisher's exact test.

d http://www.sisup​roject.fi; rs150766139.

**TABLE 2:** Family history of cancer of the identified heterozygous *NTHL1* p.Q90* (c.268C>T)^a^ carriers.

Index ID ‐cancers/tumors (age at diagnosis)	Breast/ovarian cancer(s) in first‐ and/or second‐degree relatives (age at diagnosis)	Other cancers in first‐ and/or second‐degree relatives (age at diagnosis)
Her1 ‐ Bil Br (34)	Br (u)	Stomach (u) [+], Uterus (u) [+], Lung (71), Salivary gland (u) [−], Salivary gland (u), and Lymphoma (u)
Her2 ‐ Br (38)	Ov (u)	Meningioma (u)
Her3 ‐ Br (38)	Br (62) [+]	Basal cell (50) [−], Prostate (67) [−]
Her4 ‐Br (47)	Br (64), Br (49) [−], Br (u), Br (u)	Pancreatic (50)
Her5 ‐ Br (49)	Br (65) and Brain (67), Br (42)	Adenocarcinoma^b^ (41) [‐], Renal^b^ (38) [‐]
Unsel 1 ‐ Br (79)	Br (65)	—
Unsel 2 ‐ Br (71)	Br (u)	—
Unsel 3 ‐ Br (50) and Thy (u)	Br (u)	Lung (u)
Unsel 4 ‐ Br (66)	Bil Br (45, 64)	Prostate (70)
Unsel 5 ‐ Br (59)	Br (70), Br (u), Br (u)	Esophagus (u)
Unsel 6 ‐ Br (62)	—	Renal (71)
Unsel 7 ‐ Br (58)	—	Hepatic (u)
Unsel 8 ‐ Bil Br (70)	—	Stomach (46)
Unsel 9 ‐ Br (69)	—	Throat (u)
Unsel 10 ‐ Br (62)	—	—
Unsel 11 ‐ Br (64)	—	—

Note

All tested cases marked as [+], if positive and [−], if negative for *NTHL1* p.Q90*.

Abbreviations: —, none reported; Bil Br, bilateral breast cancer; Br: breast cancer; Her, hereditary cohort; Ov, ovarian cancer; Thy, thyroid cancer; u, unknown;Unsel, unselected cohort.

a GenBank reference sequence NM_002528.5.

b Third‐degree relative, included because sample was available for *NTHL1* p.Q90* genotyping.

In the unselected breast cancer cohort, 11 *NTHL1* p.Q90* carriers were identified (11/1099, 1.0%, *p* = 0.36, OR = 0.7, 95% CI = 0.3–1.5, Table 1). The mean age at disease onset for the carriers was 64 years (range 58–79 years), which was higher than in the carriers from hereditary cohort and also higher than the mean of the unselected cohort (58 years, range 28–93 years, *p* = 0.032). Of these, five had additional breast cancer cases in their first‐ and/or second‐degree relatives (Unsel 1–5, Table 2) and seven had various other cancer types in their family (Unsel 3–9, Table 2). No samples from the relatives were available for testing.

In total, the frequency of *NTHL1* p.Q90* in the studied breast cancer cohorts (16/1333, 1.2%, *p* = 0.61, OR = 0.8, 95% CI = 0.4–1.6, Table 1) did not significantly differ from the population frequency (19/1324, 1.4%) in this geographical region. No homozygous cases were observed in any of the cohorts.

## 4 DISCUSSION

Germline loss‐of‐function variants in genes behind familial colorectal cancer and polyposis syndromes have also been reported to increase the risk for breast cancer. High lifetime breast cancer risk has been established for *STK11* and *PTEN* gene mutations that are causative for dominantly inherited hamartomatous polyposis syndromes, Peutz‐Jeghers and Cowden syndrome, respectively (Couch, Nathanson, & Offit, 2014). Also, families with more recently described polyposis predisposition syndrome caused by biallelic mutations in *MLH3* gene were reported to have extracolonic tumors, including breast cancer (Olkinuora et al., 2019). The studies from *NTHL1* families (Grolleman et al., 2019; Kuiper & Hoogerbrugge, 2015; Weren et al., 2015) add evidence for this: the same causative gene defect(s) in either recessive or dominant mode of inheritance, depending on the colorectal cancer/polyposis syndrome in concern, can also lead to predisposition to cancer in various different tissues. Whereas the evidence for breast cancer being a part of the cancer spectrum of recessively inherited *NTHL1* tumor syndrome is strong (Grolleman et al., 2019; Kuiper, Nielsen, De Voer, & Hoogerbrugge, 2020; Rivera, Castellsague, Bah, van Kempen, & Foulkes, 2015), the contribution of *NTHL1* p.Q90* heterozygosity, or even homozygosity, to breast cancer incidence in general population requires further investigation.

In the currently analyzed cohorts, heterozygous *NTHL1* p.Q90* carriers were identified in 2.1% cases with indication of hereditary predisposition to disease and in 1.0% of the breast cancer cases unselected for family history or age at disease onset. This did not significantly differ from the 1.4% carriers in the healthy population controls. Similar carrier frequencies between studied cases and the general population argue against association of *NTHL1* p.Q90* in a heterozygous state with increased breast cancer risk, although we acknowledge that the sample size of hereditary cohort is limited to 234 cases. The absence of homozygotes can also be explained by sample size of the current cohorts, but nevertheless *NTHL1* p.Q90* homozygosity appears to be an extremely rare event in cases unselected for the family history of adenomatous polyposis and colorectal cancer. It is noted that current study is limited to investigating only the germline DNA of the patients, and the presence of somatic inactivation of the *NTHL1* wild‐type allele, and the mutational signature 30 associated with biallelic loss of *NTHL1* (Grolleman et al., 2019) in patient tumors, was not addressed.

In conclusion, the current results indicate that *NTHL1* p.Q90* allele is unlikely to be a significant contributor to breast cancer risk at the population level and that the risk is not increased in heterozygous carriers, which is in line with results obtained from other cancer types (Belhadj et al., 2019). This result is particularly important for the genetic counseling units in clinical diagnostics, as the use of large gene panels containing a variety of hereditary cancer genes has become routine, even when the patients lack the classical clinical features associated with some of the genes.

## CONFLICT OF INTEREST

The authors declare that there is no conflict of interest.

## AUTHOR CONTRIBUTIONS

Katri Pylkäs, Tuomo Mantere, Anna Tervasmäki, and Timo Kumpula conceived the study. Laura Huilaja, Kaisa Tasanen, Robert Winqvist, Richarda M. de Voer, and Katri Pylkäs provided the study samples. Timo Kumpula, Anna Tervasmäki, Tuomo Mantere, and Susanna Koivuluoma performed the experiments and data analysis, supervised by Katri Pylkäs. Timo Kumpula, Anna Tervasmäki, Tuomo Mantere, and Katri Pylkäs wrote the manuscript, and all the authors read and approved the final manuscript.

## Data Availability

The data to support the findings of this study is available on request from the corresponding author.
